# Recyclable Wind Turbine Blades: A Life Cycle Analysis

**DOI:** 10.3390/ma18163762

**Published:** 2025-08-11

**Authors:** Navid Farazmandnia, Adrian Ilinca

**Affiliations:** Department of Mechanical Engineering, École de Technologie Supérieure, Montreal, QC H3C 1K3, Canada; navid.farazmandnia.1@ens.etsmtl.ca

**Keywords:** wind turbine blade materials, embodied energy, carbon footprint, simplified life cycle assessment

## Abstract

The shift towards renewable energy has highlighted the importance of sustainable practices in wind power development, particularly concerning the end-of-life (EoL) management of wind turbine blades. Conventional blades made from thermoset resins present significant recycling challenges due to their cross-linked structure, which often leads to landfill disposal or energy-intensive recycling processes. This study evaluates the environmental impacts of 45 m wind turbine blades using the Eco Audit approach across four primary life cycle stages: material production, manufacturing, transportation, and operation and maintenance. Six blade models with different fiber and resin configurations are assessed, focusing on a comparison between conventional thermoset resins and Elium, a newly developed liquid thermoplastic resin by Arkema. Elium offers promising recyclability options, including mechanical and chemical processes, which could substantially lower the environmental burden. Compared to composites made with thermoset resins, Elium-based blades demonstrate up to a 22.5% reduction in embodied energy and a 16% decrease in carbon footprint. Additionally, Elium’s compatibility with existing manufacturing processes, room-temperature curing capability, and lower processing energy contribute to its industrial feasibility. Notably, the analysis reveals that the material production phase significantly contributes to the total environmental impact, accounting for up to 98% of the embodied energy and carbon footprint in certain blade models, underscoring the importance of selecting a more sustainable resin, such as Elium, from the outset to reduce the overall environmental load.

## 1. Introduction

In recent decades, global warming and rising energy demands have driven a global shift toward renewable energy, with wind power emerging as a key solution. Wind power is gaining increasing attention in the global energy market due to its environmental and economic benefits [1]. It is also widely used for electricity generation as a clean energy technology, supported by governmental incentives and tax benefits worldwide [2].

However, a critical question remains. Are wind turbines truly as environmentally friendly as they seem throughout their whole life cycle? A significant concern arises in the end-of-life (EoL) management of wind turbine blades. Typically constructed using glass and/or carbon fiber-reinforced thermoset resins, such as epoxy [3,4,5], these blades present significant recycling challenges due to high costs, technical complexity, and limited recovery of both resins and fibers [6,7,8].

Recycling offers the benefit of conserving energy and raw materials by enabling the reuse of existing materials. Nevertheless, for fiber-reinforced composites, recycling often results in down-cycling, producing materials with inferior properties compared to the original [9]. A key factor influencing the EoL potential of these composites is the polymer matrix type, which can be thermoset or thermoplastic. Thermoplastic composites offer the potential for remelting, reshaping, and reuse, while thermoset polymers require incineration or chemical processing to recover reinforcing fibers. Despite the recent introduction of thermoplastics into structural applications, their recyclability, particularly in large-scale systems like wind turbine blades, remains insufficiently explored in the literature. The main strategies under investigation include mechanical, chemical, and thermal recycling methods [10].

To address the limitations of conventional thermoset blades, alternative recyclable materials have been proposed as a solution. Thermoplastic resin systems represent a promising class of materials due to their potential for reheating, reshaping, and fiber–matrix separation at the end of life [8]. They also offer advantages such as enhanced fracture resistance, weldability, and shorter production cycles [11]. Recent developments in two-part acrylic-based reactive thermoplastic resin systems have introduced additional benefits, including room-temperature curing and faster manufacturing times [12], which can reduce production costs while enhancing sustainability [13].

Among these materials, Elium^®^, a liquid thermoplastic resin developed by Arkema, stands out due to its mechanical properties comparable to those of epoxy, its low glass transition temperature, and its compatibility with existing fiber-processing infrastructure [14]. It cures rapidly within one hour at room temperature or five minutes at 80 °C [15]. It enables complete dissolution at room temperature within 24 h, allowing the recovery of both intact resin and reinforcing fibers [6].

While several recent studies have addressed the mechanical performance and processing advantages of Elium-based composites [15,16,17], a clear gap remains in understanding their environmental impacts compared to conventional thermoset alternatives. Most of the existing LCA studies in this area, such as Liu et al. (2019) [18] and Morini et al. (2021) [13], focus mainly on thermoset composites, often using simplified or partial bills of materials (BoMs) that omit auxiliary and consumable materials. Merugula et al. (2012) [19] evaluate reinforced blade systems, but do not address emerging thermoplastic technologies. Other works, such as Cousins et al. (2018) [7], focus on mechanical recycling, but do not address practical trade-offs or detailed BoM variability.

A notable gap in the literature is that, although many studies have investigated the properties and even the environmental impacts of thermoplastic resins such as Elium, there is a lack of direct, harmonized comparisons between Elium and conventional thermoset systems, specifically at the blade scale for wind turbine applications. Most existing LCA works either focus on material-level assessments, restrict their analysis to fiber and resin content, or overlook realistic blade-scale variability and the inclusion of auxiliary materials. Furthermore, many prior works do not fully report material assumptions, data sources, or system boundaries, which reduces their transparency and reproducibility.

This study addresses these gaps by presenting a comparative environmental assessment of wind turbine blades manufactured from both thermoset and thermoplastic (Elium-based) composites using an eco-audit LCA approach. The original contributions include the following:

- Modeling the cradle-to-grave environmental performance of six full-scale 45 m wind turbine blades using detailed, open-source bills of materials that include auxiliary and consumable materials;
- Using consistent environmental indicators (embodied energy and carbon footprint) and up-to-date, industry-relevant data sources for all material and process modeling;
- Offering a systematic, side-by-side environmental comparison of full-scale wind turbine blades produced with both conventional thermoset and emerging thermoplastic (Elium-based) technologies, thereby filling a key gap in the literature and establishing a benchmark for future comparative studies;
- Providing evidence-based recommendations for material selection in line with circular economy principles.

This work contributes to the literature by quantifying the environmental benefits and limitations of recyclable thermoplastic composites in the wind energy sector, offering actionable insights for sustainable design and policy development.

## 2. Methodology and Blade Configuration Scenarios

Life cycle assessment (LCA) is a systematic approach used to evaluate the environmental impacts of a product throughout its entire life cycle, encompassing raw material extraction, manufacturing, transportation, use, and end-of-life (EoL) management. In the context of wind turbine blades (WTBs), Ashby introduced an eco-audit methodology [20], a simplified form of life cycle assessment (LCA) that focuses on energy consumption and CO_2_ emissions, enabling rapid comparison of various blade production routes [18]. These studies concluded that the environmental favorability of a blade depends significantly on the type of resin and fiber used [21].

While previous LCA studies often focus solely on the resin and fiber content, this can lead to incomplete or overly simplified conclusions. In contrast, the present study includes a detailed bill of materials (BoM) for six full-scale 45 m blade configurations, incorporating structural and auxiliary materials to more accurately capture embodied energy and carbon impacts. These configurations differ in resin type (thermoset epoxy vs. thermoplastic Elium) and reinforcement (glass fiber, carbon fiber, or hybrid), offering a comprehensive comparison of legacy and next-generation designs.

The environmental impacts of blades made with thermoplastic Elium (Arkema, Paris, France) resin reinforced with glass or carbon fibers are compared with traditional epoxy-based thermoset blades. The objective is to evaluate their performance in terms of embodied energy and carbon footprint using consistent modeling parameters.

To assess environmental impacts, this study employs an eco-audit approach [22] that covers material production, blade manufacturing, transportation, and operation and maintenance phases. While this approach omits detailed end-of-life treatment scenarios in this section, it provides a robust baseline comparison of embedded impacts in the production and use phases.

Implementation is carried out using Ansys Granta Selector 2025 R1 [23], which supports material selection, process modeling, and environmental impact estimation across multiple lifecycle stages. Although the tool is not a substitute for a comprehensive LCA with midpoint and endpoint indicators, it provides fast, decision-oriented insight aligned with the eco-audit methodology.

The system boundary includes the following:

- The manufacturing facility and materials processing;
- Blade assembly and finishing steps;
- Transportation to the wind farm;
- Use-phase energy impacts (if modeled or assumed).

### 2.1. Blade Design Models and Adjustments

Due to the limited public access to up-to-date BoMs for current wind turbine blades, this study adapts a well-documented, earlier model featuring a hybrid configuration of glass fiber (GF) and carbon fiber (CF), denoted here as TS Hybrid. From this base, five additional blade models are derived as follows:

- TS-GF: thermoset with full glass fiber reinforcement;
- TS-CF: thermoset with full carbon fiber reinforcement;
- TP-GF: thermoplastic (Elium) with full glass fiber;
- TP-CF: thermoplastic with full carbon fiber;
- TP-Hybrid: thermoplastic with hybrid GF/CF reinforcement.

These six blade configurations are detailed in Table 1.

While the blade length of 45 m corresponds to a typical onshore turbine in the 1.5–2 MW class, it is important to emphasize that the specific blade size does not affect the comparative outcomes of this study. The main objective is to evaluate and compare the environmental impacts, particularly embodied energy and carbon footprint, of different material configurations, which are scalable and remain valid across various blade sizes.

To generate the full GF and CF models, fiber content substitutions were performed using density-based scaling. Specifically, each 1 kg of glass fiber was replaced with 0.7 kg of carbon fiber, preserving comparable structural performance. This assumption allowed for consistent comparison across uniform blade geometries. Table 2 provides the density values applied in these conversions.

Carbon fiber offers superior mechanical properties and contributes to overall blade weight reduction, potentially improving wind turbine performance. However, it carries significant environmental and economic drawbacks: the energy consumption for CFRP production is approximately 5.5 times higher than for GFRP, and material costs range from USD 50–105/kg for CF versus USD 1.5–3.5/kg for GF. Consequently, entirely CFRP blades are rare; industry practice typically uses carbon fiber selectively in structurally critical regions (e.g., the spar cap), while relying on glass fiber in lower-load regions to reduce cost and environmental burdens [28].

Another important reason for the limited use of carbon fiber in full blades is the current lack of highly efficient manufacturing techniques for large-scale carbon-fiber-reinforced polymer (CFRP) structures, which limits commercial viability and scalability.

To contextualize the material choices used in our blade models, we report resin-level properties for Elium 188 (thermoplastic) and SR 1710/SD 7820 epoxy (thermoset) in Table 3. A harmonized composite-level dataset with identical fibers and processing was not found in the literature; therefore, a resin-level comparison is the most transparent way to isolate matrix effects while keeping fibers as controlled variables in our scenarios.

### 2.2. Case Study Site: Rimouski Wind Farm Region

To accurately evaluate transportation-related environmental impacts in the life cycle assessment (LCA) of wind turbine blades, a representative site must be selected. This study adopts Rimouski, located in the Bas-Saint-Laurent region of Québec, Canada, as the reference location for the hypothetical wind farm installation.

**Figure 1:**
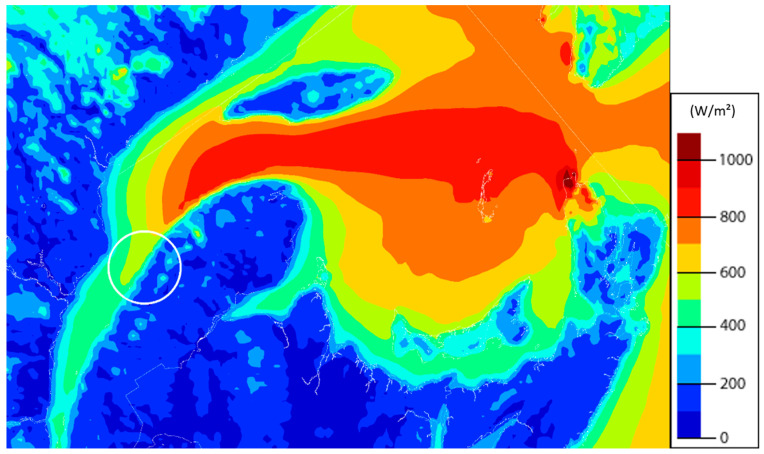
Mean annual wind energy potential at 50 m above ground-level in the Rimouski region [30].

Strategically situated at the mouth of the Rimouski River on the south shore of the St. Lawrence Estuary, Rimouski offers several advantages for wind power deployment. These include the following:

- Consistent wind resources: the regional wind atlas identifies the Rimouski area as having favorable average wind speeds at 50 m elevation, making it suitable for utility-scale wind energy generation (see Figure 1).
- Accessibility to transport logistics: proximity to waterways facilitates the delivery of large components, such as wind turbine blades, while road infrastructure allows inland transportation.
- Existing infrastructure: the presence of nearby operational wind farms confirms the feasibility of wind energy development in this location.

By anchoring the transportation and installation phases of the LCA to the Rimouski region, the study captures geographically realistic environmental burdens, particularly in terms of logistics-related embodied energy and emissions.

## 3. Environmental Performance Indicators and Material Impact Data

To evaluate the environmental performance of the six wind turbine blade (WTB) configurations, two key metrics are employed in this study: embodied energy and carbon footprint. These indicators capture the cumulative environmental impact associated with the material and process choices from raw material extraction through to production and use.

### 3.1. Embodied Energy

Embodied energy refers to the total energy required to extract, process, manufacture, and deliver a material or product. It is typically expressed in megajoules per kilogram (MJ/kg) and provides insight into the upstream energy burden associated with different material choices. Although some definitions of embodied energy extend to cover transport and end-of-life stages, the values used here pertain specifically to the production stage of the material life cycle.

Table 4 summarizes the reported ranges for the embodied energy of common composite reinforcement fibers. Considerable variability is observed in the literature, particularly for carbon fiber (CF) [31], with reported values ranging from 286 MJ/kg to over 1100 MJ/kg [20,28,29,30,31]. These discrepancies are due to differences in manufacturing technologies, data reporting practices, and material grades. The wide range also reflects ongoing improvements in carbon fiber manufacturing efficiency over the past decades. For example, embodied energy values for CF have declined from approximately 478 MJ/kg in 1999 to 286 MJ/kg in 2004, suggesting a trend toward lower impact as production scales up [28].

Despite its high energy content, carbon fiber remains attractive due to its superior mechanical properties and potential for lightweighting, which can enhance turbine performance throughout its operational life cycle. Nonetheless, the large range in reported values highlights the importance of using harmonized data sources for comparative studies.

Therefore, to ensure consistency, this study adopts the average values from Ansys Granta Selector 2025 [20], a well-documented and open-source database. These values, listed in Table 5, are used uniformly across all scenarios in the analysis. WTB manufacturers utilize E-glass and/or S-glass fibers in conjunction with resin. Despite being pricier, S-glass boasts approximately 40% more strength than E-glass of the same weight.

### 3.2. Carbon Footprint

Carbon dioxide (CO_2_) is the most prevalent anthropogenic greenhouse gas, which is mainly released from fossil fuel combustion for electricity, transportation, and industrial processes. However, other greenhouse gases, such as methane (CH_4_) and nitrous oxide (N_2_O), also contribute significantly to global warming. To unify these effects, emissions are typically reported in carbon dioxide equivalents (CO_2_-eq), which quantify the warming potential of various gases relative to CO_2_ over a standard time horizon (typically 100 years).

In material life cycle analysis, a strong correlation often exists between a material’s embodied energy and its CO_2_-eq emissions, especially in energy-intensive sectors like composites manufacturing. The values reported in Table 5 represent cradle-to-gate emissions and serve as the foundation for estimating the carbon footprint of each blade configuration assessed in this study.

## 4. Results and Discussion

The environmental performance of a wind turbine blade is evaluated by quantifying its impact across six distinct life cycle stages: (i) material production, (ii) manufacturing, (iii) transportation, (iv) operation and maintenance (O&M), (v) disposal, and (vi) end-of-life (EoL) potential. Each phase contributes uniquely to the total embodied energy and carbon footprint of the blade. To estimate the total lifetime environmental impact, the contributions from each stage are aggregated using the following expression:

E_total_ = E_material_ + E_manufacturing_ + E_transportation_ + E_O&M_ + E_disposal_ + E_EoL potential_

This comprehensive framework allows for a granular analysis of each phase, highlighting critical areas where design choices and material substitutions can yield substantial environmental benefits. Notably, the inclusion of the EoL potential term, which accounts for energy and emissions credits associated with recycling or energy recovery, ensures that circular economy considerations are integrated into the overall assessment.

### 4.1. Material

**Figure 2:**
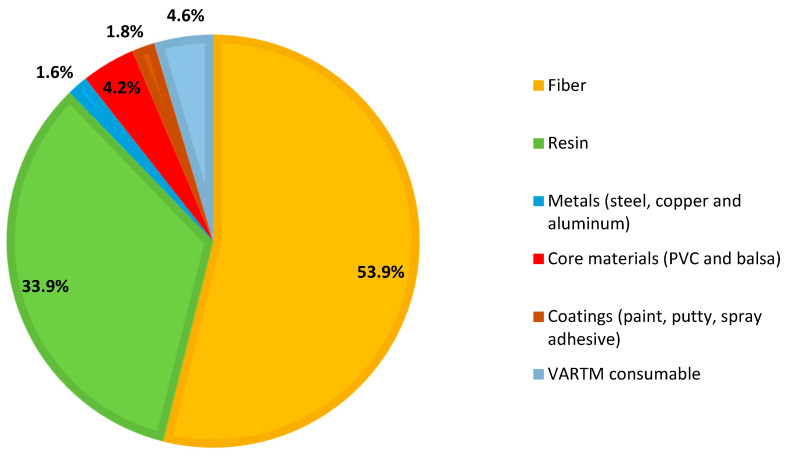
The material composition of the thermoset–glass fiber (TS-GF) wind turbine blade is expressed as a percentage of total blade weight. The chart highlights the dominance of resin and fiber compared to other materials.

The material phase represents the most substantial contribution to the overall environmental impact of wind turbine blades. To quantify this impact, the embodied energy and carbon footprint of each material used in the blade were calculated by multiplying its mass by the corresponding unit values. Figure 2 illustrates the material composition of the thermoset glass fiber (TS-GF) blade by weight. As shown, the dominant materials are fibers (E-glass and S-glass) and epoxy resin, which together constitute over 87% of the blade’s mass. All remaining materials, including balsa wood, PVC foam, steel, aluminum, and coatings, account for less than 13% combined.

### 4.2. Manufacture

The environmental impact associated with the manufacturing phase is calculated by multiplying the quantity of each processed material by its corresponding unit energy consumption and carbon footprint. The specific values for embodied energy and CO_2_ emissions vary depending on the manufacturing method employed. Table 6 summarizes the typical ranges of embodied energy and carbon footprint for several commonly used composite fabrication techniques.

Several manufacturing techniques are available for wind turbine blade production, including autoclave molding, resin transfer molding (RTM), and vacuum-assisted resin transfer molding (VARTM), also known as vacuum-assisted resin infusion (VARI). VARTM is particularly well-suited for large composite structures due to its lower processing energy and cost-effectiveness.

Most secondary materials used in blade construction, such as balsa cores, foams, or metallic fasteners, are delivered pre-processed and require no further transformation on-site. Therefore, the manufacturing-related environmental impacts considered in this study pertain solely to the composite layup and molding processes.

Following Mishnaevsky et al. [39], who note that the majority of commercial wind turbine blades are manufactured using the VARTM method, this study adopts VARTM as the exclusive manufacturing technique in all calculations.

In this study, Elium was cured using VARTM under the following assumed processing conditions: resin infusion was performed at 25 °C, with a maintained vacuum level of –950 mbar, and gel time of approximately 75 min using 3 phr dibenzoyl peroxide as the initiator. A post-curing step of 2 h at 80 °C was also applied to ensure full polymerization and mechanical performance.

For thermoset blades, the VARTM process was modeled using standard curing conditions that are representative of epoxy-based composite manufacturing. The infusion was conducted under a maintained vacuum of 1 bar below atmospheric pressure. The laminate was heated at a controlled rate of 0.5 °C per minute until reaching 50 °C and held at this temperature for 5 h. The temperature was then increased to 80 °C and maintained for 8 h to complete the curing.

### 4.3. Transportation

The transportation of wind turbine blades is typically carried out using medium- to large-sized trucks or cargo vessels, depending on the dimensions and weight of the blades, as well as the geographic location of the wind farm. While early-generation blades generally weighed less than 14 tons, recent advancements in aerodynamic performance and structural design have resulted in significantly larger and heavier blades, often exceeding 60 tons in weight.

Despite the increase in blade mass and length, conventional transportation methods remain in use, with standard road freight relying on trucks rated for 14- or 32-ton payloads. Due to the oversized dimensions of the wind turbine blades, each truck is usually limited to carrying a single blade per trip. In the absence of detailed energy consumption data for specialized blade transport trucks, it is assumed that their energy use is comparable to that of a standard 32-ton EURO 5 truck, which consumes approximately 15.2 MJ/km.

It is acknowledged that such trucks do not operate at full payload capacity. Although variations in fuel efficiency exist between fully and partially loaded conditions, these differences are difficult to quantify with precision. Therefore, a constant fuel consumption rate is assumed for environmental impact calculations.

To minimize logistical costs and emissions, manufacturers often locate blade production facilities near major wind resource regions or intended installation sites. In this study, the system boundary includes transportation from the manufacturing plant to the wind farm site, but excludes upstream raw material transport to the plant. The reference scenario assumes that LM wind power blades are manufactured in Gaspé, Québec, and transported by road over a distance of 382 km to the Rimouski Wind Farm, using a single 32-ton EURO 5 truck per blade.

It is recognized, however, that localized manufacturing is not always feasible, and in many cases, long-distance transport remains necessary to meet deployment schedules and demand.

### 4.4. Operation and Maintenance

Understanding the environmental implications of wind turbine operation and maintenance (O&M) is crucial for conducting a comprehensive life cycle assessment. Material consumption during this stage is primarily driven by two factors: routine maintenance and repairs resulting from accidental damage.

Routine maintenance, including regular inspections, minor surface repairs, and cleaning, typically involves minimal material usage and is, therefore, considered to have a negligible environmental impact. However, unplanned maintenance arising from accidental damage represents a significantly more impactful contributor. Approximately 1–3% of blades experience failure during their operational lifetime, particularly within the first ten years, often due to extreme weather conditions, structural fatigue, or operational errors. These incidents may necessitate major repairs or complete blade replacement, generating what is referred to as accidentally damaged blade waste.

From an economic perspective, O&M costs remain a substantial component of wind farm operation. In 2019, global expenditures for onshore wind farm O&M services were estimated at USD 15 billion, with unplanned repairs accounting for nearly 57% of that total [40]. With an estimated 700,000 blades in operation globally, this translates to roughly 3800 failure incidents annually, underscoring the operational challenges and environmental burden associated with maintaining these blades.

To quantify the environmental impact of this phase, based on prior studies, it is assumed that approximately 2.7% of a blade’s total mass is consumed over its service life in the form of repair materials for both scheduled and unscheduled maintenance events [41]. These repairs primarily target the outer structural layers of the blade, which are typically composed of composite materials made from fibers and resin.

According to the bill of materials (BoM) of the TS-GF (thermoset glass fiber) blade, the fiber-to-resin ratio is approximately 65:35. This composition is used to calculate the material quantities associated with O&M. As with previous life cycle stages, the environmental impact for this phase is determined by multiplying the quantity of repair material used by its respective embodied energy and carbon footprint values.

### 4.5. Disposal and End-of-Life Potential

Proper management of wind turbine blades at the end of their service life is essential for a comprehensive and accurate environmental assessment. Among the most challenging components to manage are blades made of thermoset composites. Due to their irreversible cross-linked polymer structure, thermoset materials cannot be remelted or reshaped, making them inherently more difficult to recycle than thermoplastics [42]. Consequently, there are currently no widely adopted industrial-scale recycling technologies for thermoset composite blades.

**Figure 3:**
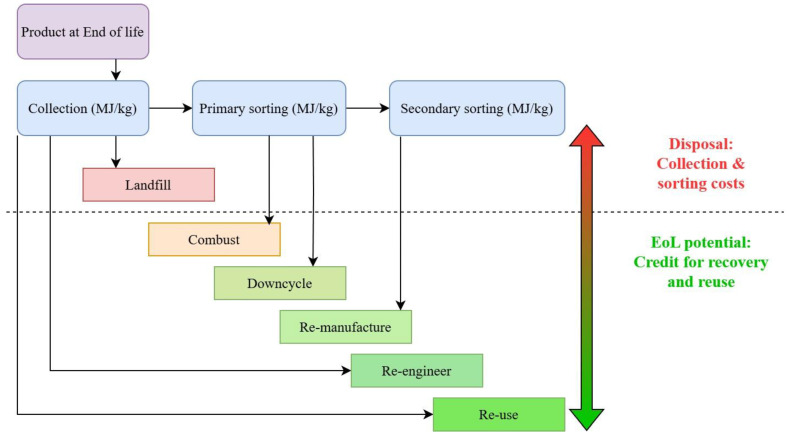
Waste management hierarchy applied to composite materials, ordered by environmental desirability. Routes range from landfill (least desirable) to reuse (most desirable), with recycling options such as downcycling and re-manufacturing in between.

In response to increasing environmental awareness and regulatory pressures, waste management strategies have become a key focus in many jurisdictions. The hierarchy of preferred waste management routes is well-established and typically follows a progression from least to most environmentally desirable options: landfill, combustion, downcycling, re-manufacturing, re-engineering, and reuse. This hierarchy, applied to composite materials, is illustrated in Figure 3.

From a life cycle perspective, the environmental impacts associated with the end-of-life phase can be broken down into two components: disposal and end-of-life (EoL) potential.

- Disposal encompasses all activities required to manage a blade at the end of its functional life. This includes the collection and transportation of the blade, separation, and sorting of materials, and, when applicable, final disposal in landfills or incineration.
- EoL potential refers to the environmental credits or offsets that can be attributed to the recovery and reuse of materials in subsequent product life cycles. These credits are conditional on both the recycling method employed and the material composition. While EoL potential does not reduce the immediate environmental burden of the current product, it contributes to a broader reduction in lifecycle impacts by displacing the need for virgin materials in future production cycles.

Because these EoL credits fall outside the conventional system boundaries of the current product life cycle, they are reported separately in the analysis. Nonetheless, they represent a key opportunity to enhance the sustainability of wind turbine blades, particularly when recyclable materials, such as thermoplastics, are utilized.

### 4.6. Lifetime Impact

As discussed in previous sections, the total lifetime environmental impact of a wind turbine blade is calculated by aggregating the contributions from the following six life cycle phases: material production, manufacturing, transportation, operation and maintenance (O&M), disposal, and end-of-life (EoL) potential. Table 7 presents a detailed breakdown of embodied energy and climate change impact (in terms of CO_2_-equivalent emissions) for the baseline configuration, namely the thermoset–glass fiber (TS-GF) blade.

The results in Table 6 confirm that the material and manufacturing stages dominate the environmental impact, together accounting for over 96% of the total embodied energy and more than 97% of the CO_2_-equivalent emissions.

While the transportation of raw materials to the manufacturing site is excluded from the system boundary, the transport of finished blades to the wind farm site is included. This transport phase represents less than 1% of the total environmental impact, and is therefore considered negligible. Similarly, the O&M phase contributes just 2% of the total embodied energy and less than 1% of the carbon footprint. Despite manufacturing accounting for approximately 11% of the embodied energy, its contribution is constrained by the limited range of low-impact composite fabrication methods available for large blades. Among these, vacuum-assisted resin transfer molding (VARTM) is one of the least energy-intensive options and is used in our model.

Disposal and EoL contributions are treated separately. The disposal phase encompasses the environmental costs associated with collecting and processing the blade at the end of its useful life. Meanwhile, EoL potential is considered an environmental credit, reflecting the avoided impact from partial material recovery. Because industrial recycling solutions for thermoset composites are not yet commercially viable [43], mechanical downcycling was selected as the EoL strategy. In this scenario, approximately 55% of the original glass fiber can be recovered, albeit with diminished mechanical performance [44].

**Figure 4:**
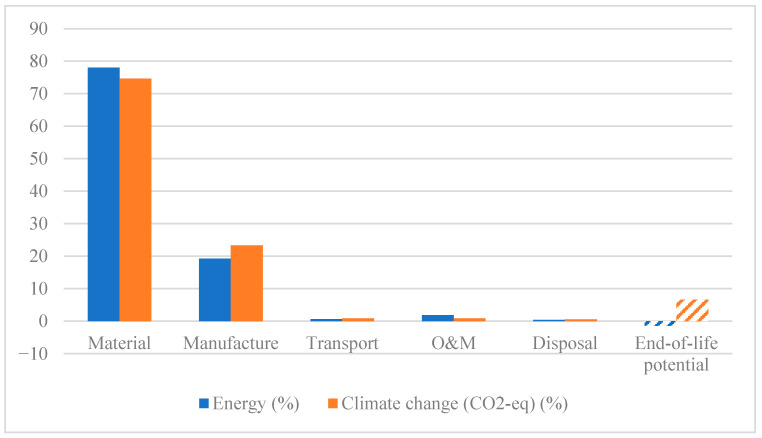
Relative contribution (%) of each life cycle phase, materials, manufacturing, transportation, operation and maintenance (O&M), disposal, and end-of-life (EoL), potential to the total environmental impact of the TS-GF wind turbine blade.

**Figure 5:**
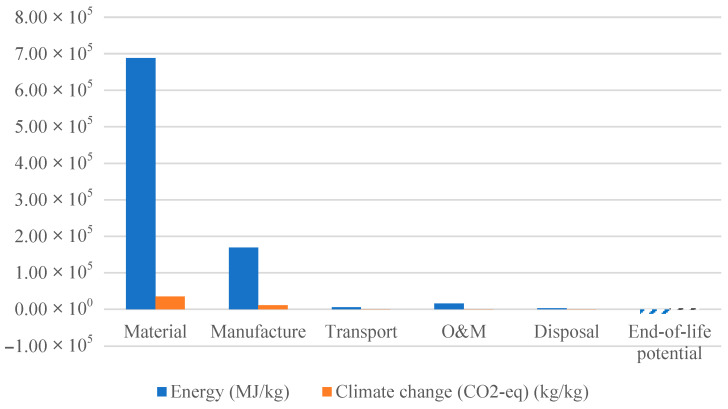
Absolute environmental impacts of each life cycle phase, materials, manufacturing, transportation, operation and maintenance (O&M), disposal, and end-of-life (EoL), potential to the total environmental impact of the TS-GF wind turbine blade.

Figure 4 illustrates the relative contribution of each life phase to the total environmental impact of the TS-GF blade. To complement this, Figure 5 presents the absolute values of embodied energy and carbon footprint for each stage, allowing for a more detailed and quantitative comparison.

## 5. Discussion

To reduce the total environmental impact of wind turbine blades, special attention must be paid to materials that either (1) lower embodied energy and carbon footprint or (2) enhance end-of-life (EoL) recovery potential. While prior studies have examined the environmental impact of various thermoset composites [13], this work shifts the focus toward thermoplastic resins, specifically Elium 188, which is used in combination with glass, carbon, or hybrid fibers.

### 5.1. Quantitative Evaluation of the Environmental Advantages of Thermoplastic Blades

**Figure 6:**
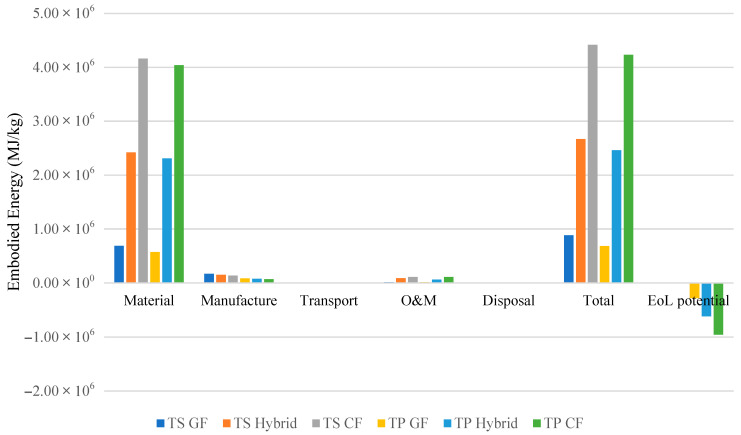
Comparison of embodied energy across six wind turbine blade configurations using thermoset (TS) and thermoplastic (TP) resins with various fiber types (glass, carbon, and hybrid). Results are shown in megajoules (MJ).

**Figure 7:**
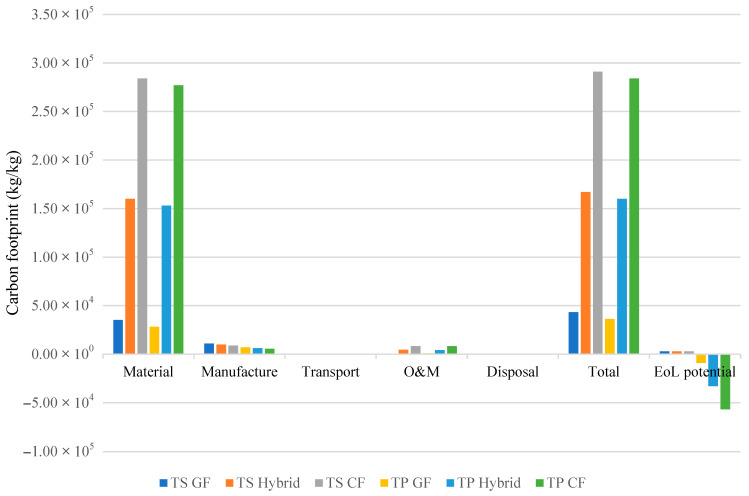
Carbon footprint of six wind turbine blade configurations composed of thermoset (TS) and thermoplastic (TP) resins with glass, carbon, or hybrid fibers. Results are expressed in kilograms of CO_2_-equivalent emissions per kilogram of blade.

As previously discussed, one of the key advantages of thermoplastic composites lies in their recyclability, which allows for a significant increase in EoL potential credit. In contrast, due to their irreversible cross-linked structures, thermoset resins offer limited recycling pathways. The comparative results for six different blade configurations, three based on thermosets (TS) and three on thermoplastics (TP), are presented in Table 8 and visualized in Figure 6 and Figure 7.

These results show that thermoplastic blades consistently outperform thermoset counterparts in terms of overall sustainability. For instance,

- TP-GF blades exhibit 22.4% lower embodied energy and 22.2% lower carbon footprint than TS-GF blades.
- TP-Hybrid and TP-CF configurations also exhibit improvements, albeit less pronounced, with embodied energy reductions of 7.9% and 4.3%, respectively, and carbon footprint reductions of 6.3% and 3.3%.

Beyond initial production impacts, thermoplastic composites offer higher EoL recovery. Their ability to be reprocessed without degradation of mechanical properties enables complete recycling into core materials. In contrast, recycling carbon fibers from thermoset composites remains energy-intensive and yields fibers with diminished strength [18].

### 5.2. Trade-Off Between Mechanical Performance and Environmental Impact in Material Selection

Both thermoplastic and thermoset carbon-fiber-reinforced composites present a significant sustainability challenge despite their mechanical advantages. Thermoset (TS) and thermoplastic (TP) carbon fiber composites exhibit markedly higher environmental footprints than alternative configurations:

- TP-CF compared to TP-Hybrid has 72% and 77.5% higher embodied energy and carbon footprint, respectively.
- TP-CF compared to TP-GF has 518% and 692% higher embodied energy and carbon footprint, respectively.
- TS-CF compared to TS-Hybrid has 65.6% and 74.3% higher embodied energy and carbon footprint, respectively.
- TS-CF compared to TS-GF has 401% and 573% higher embodied energy and carbon footprint, respectively.

These figures highlight a critical tension; while carbon fiber delivers superior mechanical properties, its environmental cost is disproportionately high.

These numbers stem from carbon fiber’s extreme production intensity (959 MJ/kg vs. 32.5 MJ/kg for E-glass, Table 4), which drives material-phase impacts to their highest levels in carbon-reinforced systems. Across all configurations, material production dominates lifecycle burdens at 78–95.5% of embodied energy and 74.6–95.2% of carbon footprint. Critically, carbon configurations (TP-CF/TS-CF) exhibit the most extreme material dominance, reaching 94.1–95.5% of energy use and 94.8–95.2% of emissions, while glass fiber systems show substantially lower material contributions (78.0–78.0% energy, 74.6–74.6% carbon).

The manufacturing stage remains secondary at 1.6–19.2% energy and 1.9–23.3% emissions, and is too limited to counterbalance material burdens, even with advanced processing. Concurrently, carbon fiber’s 20–30 times cost premium (USD 50–105/kg vs. 1.5–3.5/kg for GF) creates economic barriers to sustainable implementation.

### 5.3. Practicalities and Challenges of End-of-Life (EoL) Recycling

For thermoset composite wind turbine blades, the only viable end-of-life (EoL) strategies currently available at an industrial scale are landfill, mechanical recycling, and incineration. To better assess their end-of-life (EoL) potential, combustion was selected in this study, as it allows for partial energy recovery, despite the drawback of generating additional and undesirable carbon emissions.

In the case of thermoplastic composites, some studies have investigated the retained tensile strength after recycling through mechanical and chemical processes (commonly referred to as solvolysis), the two main techniques applied to Elium-based systems. The results reported so far are promising in terms of mechanical performance [45]. However, as with thermoset composites, a high amount of energy is still required to recover carbon fibers.

As a result, the EoL potential further supports the adoption of thermoplastics. For TP-GF blades, 41.2% of the total embodied energy can be recovered through recycling. This potential decreases to 25.1% and 22.6% for hybrid and carbon-fiber-reinforced models, respectively. In terms of emissions, the carbon footprint can be reduced by 24.5% (TP-GF), 20.1% (TP-Hybrid), and 19.6% (TP-CF).

However, in addition to these end-of-life benefits, particular processing and application challenges must be considered. Despite their recyclability and potential for more circular design strategies, thermoplastic composites face several technical challenges when applied to wind turbine blades. One major issue lies in the higher viscosity of thermoplastic resins, which limits their ability to fully impregnate fiber reinforcements during standard infusion processes like VARTM, potentially leading to suboptimal mechanical performance. Additionally, bonding thermoplastic matrices to core materials (e.g., balsa or foam) remains difficult due to differences in surface chemistry and the absence of cross-linking reactions, which can affect the structural integrity of sandwich components. Processing at elevated temperatures also poses equipment and energy challenges, particularly in large-scale blade manufacturing. Finally, while thermoplastics offer advantages in reshaping and welding, scaling up these technologies for multi-meter blades is still under development, with limited industrial demonstrations to date. These practical considerations must be weighed carefully against environmental benefits when evaluating TP-based blade configurations.

### 5.4. Future Prospects for Economic and Industrial Applicability

While thermoplastic blades present a compelling environmental case, cost and performance trade-offs must be strategically balanced. Carbon fibers, though strong and lightweight, carry significantly higher economic and environmental costs that may not justify their use in many applications. To optimize future blade designs, we propose this material selection framework:

1. Carbon fiber justification: reserve exclusively for performance-critical applications (e.g., offshore blades > 80 m) where mechanical benefits demonstrably outweigh sustainability costs.
2. Glass fiber prioritization: optimize eco-performance for most onshore installations through thermoplastic GF systems.
3. Hybrid implementation: employ partial carbon reinforcement only when operational benefits exceed environmental premiums by quantifiable margins.

## 6. Conclusions

This study presents a comprehensive life cycle assessment (LCA) of six wind turbine blade configurations, utilizing both thermoset and thermoplastic resins in combination with glass, carbon, or hybrid fiber reinforcements. The results offer critical insights into how material choices influence the environmental footprint of wind turbine blades across all life cycle stages, from production to end-of-life.

Our findings demonstrate that thermoplastic composites, particularly when reinforced with glass fibers, significantly outperform conventional thermoset systems in terms of both embodied energy and carbon footprint. When compared to their thermoset counterparts, thermoplastic blades exhibit up to 22.5% lower embodied energy and 22.3% lower carbon emissions, making them a more sustainable alternative. Furthermore, thermoplastics offer superior end-of-life (EoL) performance, enabling up to 41.2% energy recovery, while thermosets remain largely unrecyclable at an industrial scale.

One of the central conclusions of this research is that material selection is the single most influential factor in determining a blade’s environmental impact. On average, over 89.3% of the total embodied energy and 87.5% of the carbon footprint can be attributed to the raw materials used. This underscores the urgent need to integrate sustainability into the early stages of blade design, where material choice can significantly shape long-term environmental performance.

Although carbon fiber composites provide excellent mechanical properties, their environmental and economic drawbacks are substantial. Regardless of resin type, blades composed entirely of carbon fibers require more energy to produce and are associated with a much larger carbon footprint. Additionally, current recycling technologies for carbon fiber, particularly those derived from thermosets, remain energy-intensive and inefficient, rendering full-carbon blade designs both environmentally and commercially unsustainable.

This work makes the following novel contributions to the existing literature on wind turbine blade sustainability:

- It is one of the first studies to directly compare multiple thermoset and thermoplastic composite blade configurations using a complete cradle-to-grave life cycle assessment (LCA) framework, incorporating realistic assumptions for transportation, manufacturing, maintenance, and end-of-life treatment.
- It quantifies the EoL potential of thermoplastics in blade recycling, a frequently discussed but rarely measured advantage, by applying realistic downcycling and recovery rates to embodied energy and emissions.
- It provides a detailed benchmark for embodied energy and carbon emissions across six blade types, addressing a significant research gap in comparative life cycle assessment (LCA) data for emerging thermoplastic technologies in large-scale structural applications.
- It contributes practical insights into the trade-offs between mechanical performance and sustainability, helping guide future material choices in turbine blade design.

By filling these gaps, this research supports the development of next-generation wind turbine blades that are not only more durable and efficient but also environmentally responsible and end-of-life ready, a key priority as the wind energy sector scales up globally.

## 7. Perspectives and Directions for Future Research

While this study offers a comparative life cycle assessment of six wind turbine blade configurations, certain limitations must be acknowledged. Due to restricted access to detailed industrial data and variability in secondary sources, we were unable to conduct a formal uncertainty analysis or sensitivity study. These elements would be valuable additions to future work aiming to assess the robustness of environmental outcomes across broader scenarios. Future studies incorporating such analyses could further refine the environmental profile of advanced blade materials, particularly in relation to recycling strategies and supply chain variability.

Despite promising developments in recycling techniques for thermoset composites, most methods remain confined to laboratory or pilot-scale applications, limiting their practical implementation. Consequently, accurately quantifying the end-of-life (EoL) potential remains a challenge. Future research should aim to conduct comparative evaluations of EoL pathways for both thermoset and thermoplastic composites, including mechanical, chemical, and thermal recycling methods, to better inform life cycle strategies and policy decisions.

Moreover, while this study has focused primarily on environmental indicators, the techno-economic feasibility of adopting thermoplastic composites at scale warrants further investigation. Future studies should include a comprehensive cost–benefit analysis that compares thermoplastic and thermoset blade configurations, factoring in manufacturing costs, maintenance expenses, recyclability potential, and long-term lifecycle savings. Such analyses are crucial for supporting industrial decision-making and facilitating the broader adoption of recyclable thermoplastic materials in wind turbine blade production.

## Figures and Tables

**Table 1:** Bill of materials for six wind turbine blade models with varying fiber and resin types.

	Name	Materials	Country of Origin	TS-GF	TS-Hybrid	TS-CF	TP-GF	TP-Hybrid	TP-CF
Materials Used on Blade (kg)	Glass Fiber Unidirectional	S-glass	China	2414	1207		2414	1207	
	Glass Fiber Biaxial/Triaxial	E-glass	China	3084	1542		3084	1542	
	Carbon Fiber		Canada		1924	3848		1924	3848
	Thermoset Resin	Epoxy	USA	2940	2940	2940			
	Thermoplastic Resin	Elium 188 XO	France				2940	2940	2940
	Steel (nuts, washer)	42CrMoA		103.7	103.7	103.7	103.7	103.7	103.7
	Copper (thunder protection system)	Copper, C10200, wrought		31.5	31.5	31.5	31.5	31.5	31.5
	Aluminum	Aluminum 6010, wrought		2.5	2.5	2.5	2.5	2.5	2.5
	Balsa	End-grain balsa (0.15)		208.9	208.9	208.9	208.9	208.9	208.9
	PVC	PVC crosslinked foam, DH 0.060		156.6	156.6	156.6	156.6	156.6	156.6
	Paint	Acrylic or alkyd paint		85.4	85.4	85.4	85.4	85.4	85.4
	Putty	Acrylic wall putty		62	62	62	62	62	62
	Spray Adhesives	Synthetic elastomer-based adhesive		4.4	4.4	4.4	4.4	4.4	4.4
VARTM Consumable Materials (kg)	Continuous Filament Mat	E-glass with acid corrosion resistance		21	21	21	21	21	21
	Release Film	PA66		28.9	28.9	28.9	28.9	28.9	28.9
	Vacuum Bag Film	PA 60% PE 40%		111.3	111.3	111.3	111.3	111.3	111.3
	Porous Membrane	PTFE		9.9	9.9	9.9	9.9	9.9	9.9
	Flow Mesh Layer	Polyethylene		60	60	60	60	60	60
	Breather Bleeder	Polyester		12.8	12.8	12.8	12.8	12.8	12.8
	Vacuum Bagging Sealant Tape			65.4	65.4	65.4	65.4	65.4	65.4
	Resin Flow Runner Pipes (Spiral)	PE		28.6	28.6	28.6	28.6	28.6	28.6
	Resin Flow Runner Pipes (Omega)	PVC		1.2	1.2	1.2	1.2	1.2	1.2
	Steel Spiral Pipes	Steel		14.8	14.8	14.8	14.8	14.8	14.8
	PVC Holder	PVC		40.9	40.9	40.9	40.9	40.9	40.9
	T Fitting + Infusion Valve	PPR (random copolymer)		5	5	5	5	5	5
	Total weight of BOM (kg)			9493	8668	7843	9493	8668	7843

**Table 2:** Densities of fiber materials used for substitution in blade models.

Material	Type	Density (kg/m^3^)	Source/Notes
Glass Fiber (Unidirectional)	S-glass	2490	Typical value for high-strength S-glass [24,25]
Glass Fiber (Biaxial/Triaxial)	E-glass	2550	Commonly used in structural composites [24,25]
Carbon Fiber	PAN-based (high-modulus)	1750	Average value for aerospace-grade CF [26,27]

**Table 3:** Comparative mechanical and thermomechanical properties of composite systems [29].

Property	Elium 188	Epoxy SR 1710/SD 7820
Tensile strength (MPa)	66	78
Tensile modulus (GPa)	3.2	2.8
Elongation at break (%)	2.8	6.2
Flexural strength (MPa)	111	117
Flexural modulus (GPa)	2.9	2.8
Density (g/cm^3^)	1.01	1.15
Viscosity@25 °C (m.Pa.s)	100	350
Glass transition temperature (°C)	102	127

**Table 4:** Reported embodied energy values of selected fiber reinforcements from the literature.

Fibers	Embodied Energy (MJ/kg)	Reference
Carbon fiber	532–558	[32,33]
	286–478	[34]
	913–1013	[23]
	1100	[35]
E-glass fiber	13–32	[36]
	30.9–34	[23]
S-glass fiber	51.1–56.3	[23]
China reed fiber	3.6	[37]
Flax fiber	6.5	[36,37]

**Table 5:** Standardized environmental impact and CO_2_ emission values for materials used in this study, based on Granta Selector 2025 [23].

Material	Embodied Energy (MJ/kg)	CO_2_ Emission (kg/kg)	Water Consumption (L/kg)
Carbon fiber	959	68.1	7.4
S-glass fiber	52.7	3.36	296
E-glass fiber	32.5	2.54	94.5
Aramid fiber	257	13.1	940
Epoxy resin	132	6.5	28.0
Polyester resin	79.2	2.6	238
Steel	32.3	2.4	52.1
Aluminum	201	13.7	1210
Balsa	12.3	0.6	687
PVC	80.6	5.2	455
Paint and adhesives	89.4	5.2	295

**Table 6:** Unit embodied energy and carbon footprint for different composite manufacturing techniques.

Production Method	Embodied Energy (MJ/kg)	Carbon Footprint (kg/kg)	Reference
Wet hand layup	13.5		[38]
Prepreg	123		[38]
Resin transfer molding (RTM)	34.4–37.9	2.26–2.5	[23]
Vacuum-assisted resin transfer molding (VARTM)	28.8–31.7	1.89–2.09	[23]

**Table 7:** Embodied energy and climate change contributions for the TS-GF blade.

Phase	Energy (MJ)	Energy (%)	Climate Change (CO_2_-eq) (kg)	Climate Change (CO_2_-eq) (%)
Material	6.88 × 10^5^	78	3.52 × 10^4^	74.6
Manufacture	1.69 × 10^5^	19.2	1.1 × 10^4^	23.3
Transport	5.81 × 10^3^	0.6	370	0.8
O&M	1.61 × 10^4^	1.8	404	0.8
Disposal	3.25 × 10^3^	0.4	227	0.5
Total (for first life)	8.82 × 10^5^	100	4.72 × 10^4^	100
End-of-life potential	−1.16 × 10^4^		2.83 × 10^3^	

**Table 8:** Embodied energy and carbon footprint contributions for all six blades.

Phase	Environmental Indicators	TS-GF	TS Hybrid	TS CF	TP GF	TP Hybrid	TP CF
Material	Energy (MJ)	6.88 × 10^5^	2.42 × 10^6^	4.16 × 10^6^	5.72 × 10^5^	2.31 × 10^6^	4.04 × 10^6^
	Carbon footprint (kg)	3.52 × 10^4^	1.6 × 10^5^	2.84 × 10^5^	2.82 × 10^4^	1.53 × 10^5^	2.77 × 10^5^
Manufacture	Energy (MJ)	1.69 × 10^5^	1.52 × 10^5^	1.36 × 10^5^	8.61 × 10^4^	7.77 × 10^4^	6.92 × 10^4^
	Carbon footprint (kg)	1.1 × 10^4^	9.9 × 10^3^	8.82 × 10^3^	6.92 × 10^3^	6.24 × 10^3^	5.57 × 10^3^
Transport	Energy (MJ)	5.81 × 10^3^	5.3 × 10^3^	4.8 × 10^3^	5.81 × 10^3^	5.3 × 10^3^	4.8 × 10^3^
	Carbon footprint (kg)	370	338	306	370	338	306
O&M	Energy (MJ)	1.61 × 10^4^	8.84 × 10^4^	1.12 × 10^5^	1.56 × 10^4^	6.24 × 10^4^	1.12 × 10^5^
	Carbon footprint (kg)	404	4.59 × 10^3^	8.16 × 10^3^	907	4.19 × 10^3^	8.17 × 10^3^
Disposal	Energy (MJ)	3.25 × 10^3^	3.09 × 10^3^	2.93 × 10^3^	4.88 × 10^3^	4.73 × 10^3^	4.58 × 10^3^
	Carbon footprint (kg)	227	216	205	342	331	320
Total	Energy (MJ)	8.82 × 10^5^	2.67 × 10^6^	4.42 × 10^6^	6.84 × 10^5^	2.46 × 10^6^	4.23 × 10^6^
	Carbon footprint (kg)	4.32 × 10^4^	1.67 × 10^5^	2.91 × 10^5^	3.63 × 10^4^	1.6 × 10^5^	2.84 × 10^5^
EoL potential	Energy (MJ)	−1.16 × 10^4^	−1.16 × 10^4^	−1.16 × 10^4^	−2.82 × 105	−6.19 × 10^5^	−9.56 × 10^5^
	Carbon footprint (kg)	2.83 × 10^3^	2.83 × 10^3^	2.83 × 10^3^	−8.98 × 10^3^	−3.3 × 10^4^	−5.69 × 10^4^

## Data Availability

The original contributions presented in this study are included in the article. Further inquiries can be directed to the corresponding author.
